# After/Lives: Insights from the COVID-19 Pandemic for Gay Neighborhoods

**DOI:** 10.1007/978-3-030-66073-4_17

**Published:** 2020-11-30

**Authors:** Sam Miles, Jack Coffin, Amin Ghaziani, Daniel Baldwin Hess, Alex Bitterman

**Affiliations:** 17 Department of Architecture and Design, Alfred State University of New York, New York, USA; 18 Department of Urban and Regional Planning, University at Buffalo, Buffalo, NY USA; 19 London School of Hygiene and Tropical Medicine, London, UK; 20 University of Manchester, Manchester, UK; 21 University of British Columbia, Vancouver, BC Canada; 22 University at Buffalo, State University of New York, Buffalo, NY USA; 23 Alfred State College, State University of New York, Alfred, NY USA

**Keywords:** AIDS, Coronavirus, COVID-19, Gayborhoods, Gay neighborhoods, HIV, LGBTQ+, Pandemic, Public health

## Abstract

Beginning in 2020, COVID-19 produced shock-shifts that were felt across the globe, not least at the level of the local neighborhood. Some of these shifts have called into question the role of physical places for face-to-face gatherings, including those used by LGBTQ+ people. Such open questions are a key concern for a book on gayborhoods, so this chapter engages in three analytic tasks to provide preliminary reflections on how pandemics problematize places. While acknowledging a range of threats and challenges that the pandemic poses to the future of LGBTQ+ spaces, this chapter focuses on the potential opportunities and unexpected benefits that COVID-19 can create, running counter to more pessimistic predictions that abound in popular discourse. First, the chapter contextualizes how the COVID-19 pandemic is reminiscent of the HIV/AIDS pandemic, allowing the gayborhood to be well-equipped to respond with grassroots activism, particularly in the face of government inaction or apathy. Second, the chapter explores trends that can ensure the future vitality of LGBTQ+ spaces, including (i) the potential of mutual aid networks, (ii) the power of institutional anchors in LGBTQ+ placemaking efforts, (iii) urban changes related to homesteading and population shifts, (iv) innovations in the interior design of physical spaces, and (v) opportunities to enhance social connections through augmented virtual engagements. Far from signaling the death knell of LGBTQ+ spaces, these trends demonstrate the enduring appeal provided by neighborhoods and communities. Third, the cognitive schemas of lockdowns, re-closeting, and digitalscapes are identified as unique expressions of the shifting spatialities of sexuality in post-pandemic urban space. The chapter concludes by arguing that place will still matter for LGBTQ+ people in a post-COVID-19 era, albeit with altered meanings and material expressions. The socio-spatial consequences of the novel coronavirus will be a confluence of positive *and* negative developments, and while some will be reversed as soon as an effective vaccine is found, others will linger indelibly in bodies and the built environment for years to come.

## Introduction: Once More, Without Human Contact?

In the midst of the coronavirus epidemic, we are all bombarded… by calls not to touch others but to isolate ourselves, to maintain a proper corporeal distance. (Žižek 2020: 1)

**Fig. 17.1:**
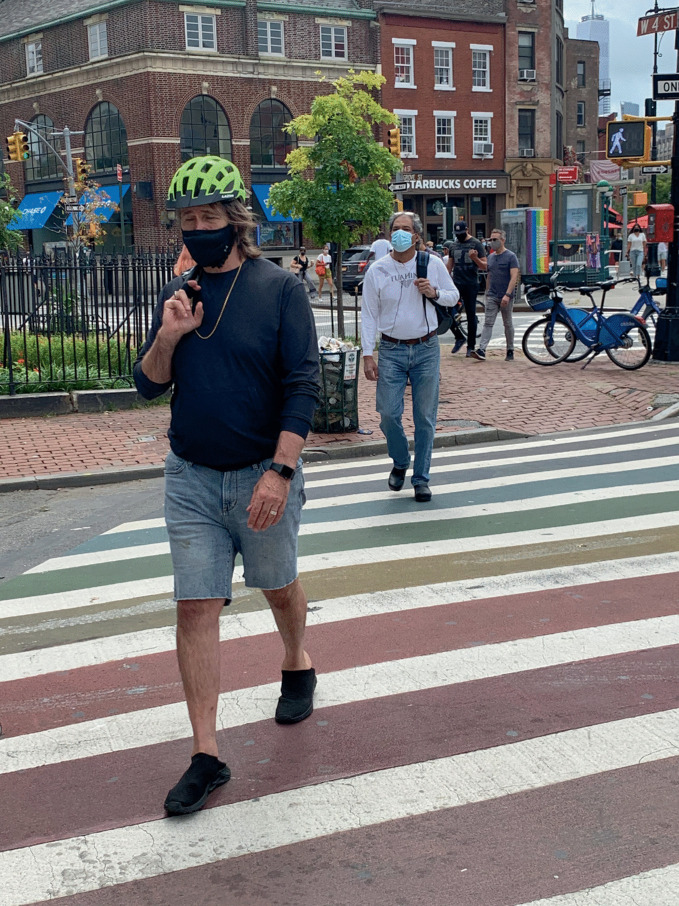
Greenwich Village is one of the key centers of LGBTQ + life in New York City (*Source* Image by Anne Hanavan)

**Fig. 17.2:**
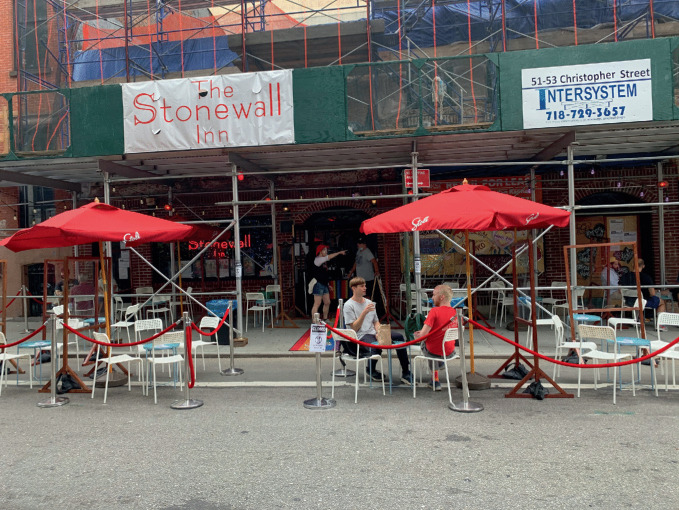
Pavement tables permit spatial distancing for customers at the famed Stonewall Inn in Greenwich Village in New York City (*Source* Image by Anne Hanavan)

During a global pandemic where people are implored to remain spatially distanced, are physical places of co-presence still viable for LGBTQ + communities? Are they important in an era of virtual meetings and online dates? How has the significance of gay neighborhoods changed with the novel coronavirus as a backdrop? These are vital questions for an edited volume that has sought to demonstrate that “gayhorhoods matter.” Several chapters have looked at LGBTQ+ districts in the past and the present, as well as speculating about their possible futures, yet they were written *before* COVID-19 spread across the globe and transformed our social, economic, and geographical realities in ways hitherto unfathomable. A critical reader might conclude that some of the preceding chapters require a reorientation to accommodate post-COVID-19 realities. In this epilogue, five contributors convene to articulate a vision that acknowledges the need to reconceptualize gayborhoods. Ultimately, we argue that places will still matter in the post-pandemic urban landscape. Far from signaling the death knell of LGBTQ + spaces and places, the collective experience with COVID-19 demonstrates the enduring appeal provided by urban areas of physical proximity (see Fig. 17.1). Quarantine measures and the “new normal” of technologically mediated meetings have threatened the economic viability of gayborhoods, but absence, especially an enforced one, makes the heart grow fonder—and thus a second wave of place attachments and localized activism present a viable future as well (see Fig. 17.2).

**Fig. 17.3:**
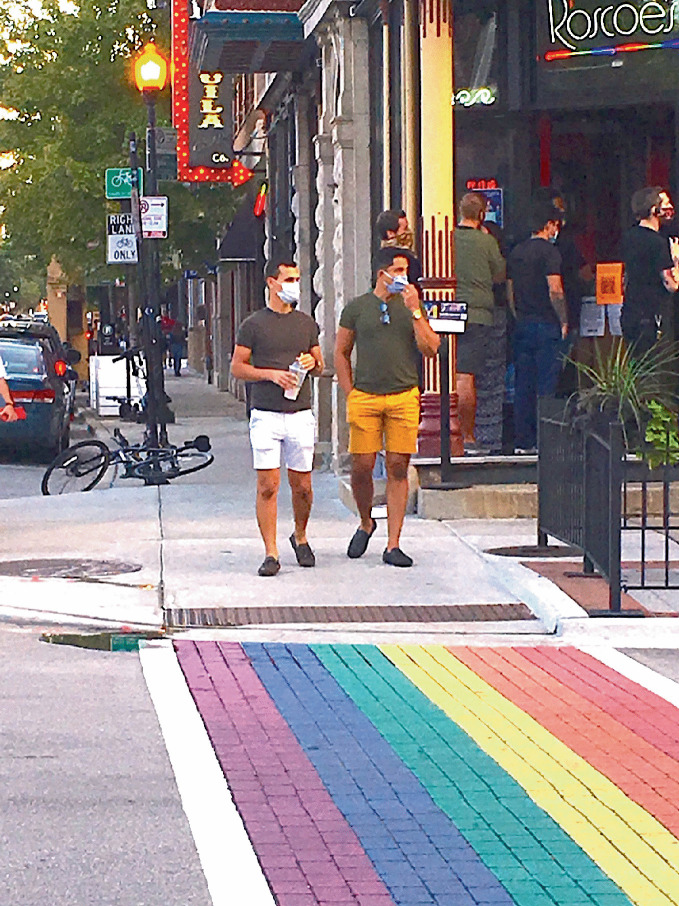
Residents and visitors in gayborhoods everywhere (in Chicago) follow COVID-19 regulations including wearing masks and practicing spatial distancing (*Source* Image by William Ivancic)

While this chapter can only speculate about the post-pandemic realities of LGBTQ + people and places, we predict that the socio-spatial consequences of the coronavirus will be a confluence of positive *and* negative developments. While some of these will be reversed as soon as an effective vaccine is found, others will linger in our bodies and in the built environment (see Fig. 17.3). For instance, the call to maintain physical distance is not so different from the injunction on intimate contact imposed by the arrival of HIV/ AIDS in the 1980s. For many LGBTQ + people, the current situation is reminiscent of the HIV/AIDS pandemic; even those too young to have experienced it first hand still grew up in its cultural shadows (Bitterman 2020b). This prior experience is productive—the gayborhood is uniquely equipped to respond with grassroots activism, particularly in the face of government inaction or apathy—but it is also potentially problematic, as it may trigger negative memories of trauma, encourage individualistic withdrawal from human contact, or provide historical models that delimit reimagining what LGBTQ + geographies could become. On this last point, while there are certainly parallels between the two pandemics, there are also significant differences. Digital technology, for example, may encourage LGBTQ + communities to diffuse, rather than to gather together in gayborhoods as they did during the HIV/ AIDS pandemic (Coffin 2021; Miles 2021).

The remainder of this chapter proceeds as follows. First, we articulate the analytic parameters of the current pandemic. In this discussion, we define COVID-19 as a “glocal” phenomenon, one with transnational as well as local expressions and implications. This property of the pandemic is important to understand before we can consider how it might affect the meanings of the gayborhood and other types of LGBTQ+ spaces. Next, we review a range of empirical transformations and trends. We seek to maintain a balanced tone in our discussion, considering the threats and challenges that COVID-19 poses alongside opportunities and unexpected benefits for the future of LGBTQ + urban spaces. Based on this discussion, we propose that academics, activists, and other stakeholders should reconsider the guiding metaphor of “the gayborhood” and instead conceptualize LGBTQ+ geographies in more innovative and expansive ways.

## Do Places Matter? Empirical Trends for the Future of LGBTQ+ Spaces

**Fig. 17.4:**
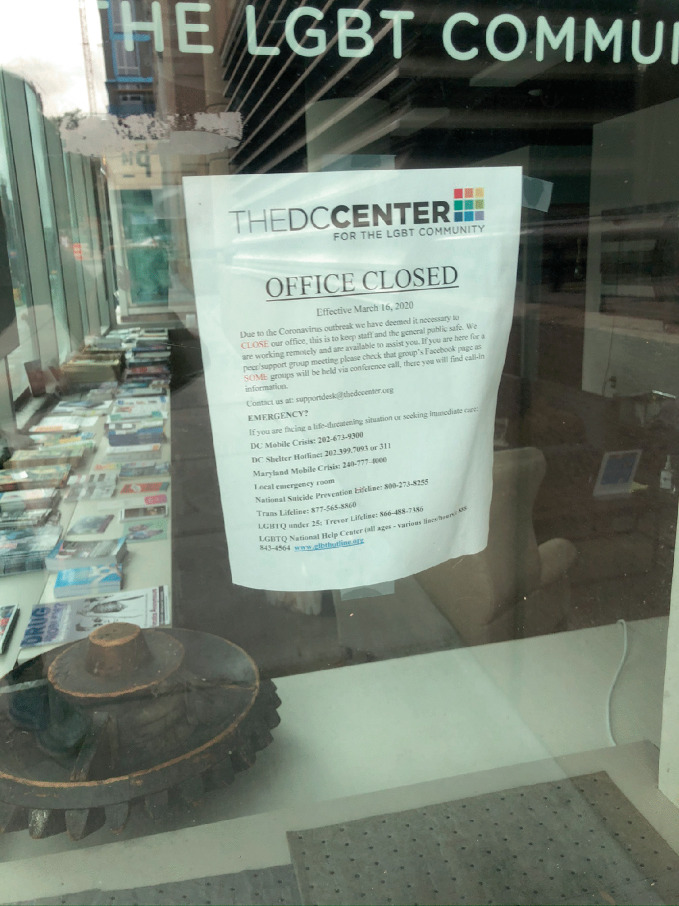
Community organization and non-profit services adapted to meet COVID-19-related regulations, including the DC Center for LGBT Community in Washington, DC (*Source* Image by Elizabeth R. June)

It is limiting to view COVID-19 as a uniformly destructive force for gayborhoods and other types of LGBTQ + spaces. In this section, we highlight the unexpected opportunities that a pandemic can generate for urban sexual communities. Five trends strike us as promising, even in moments of widespread uncertainty, for ensuring the vitality of LGBTQ+ spaces: (1) the power of mutual aid networks, (2) the power of institutional anchors in placemaking efforts, (3) urban change related to homesteading and population shifts, (4) innovations in the architecture and interior design of physical spaces, and (5) opportunities to enhance social connection through augmented virtual engagements. Together, these themes can refocus conversations about the effects of pandemics away from assumptions of demise and community dilution to emergent empirical realities of reconstitution and community resilience (see Fig. 17.4).

**Fig. 17.5:**
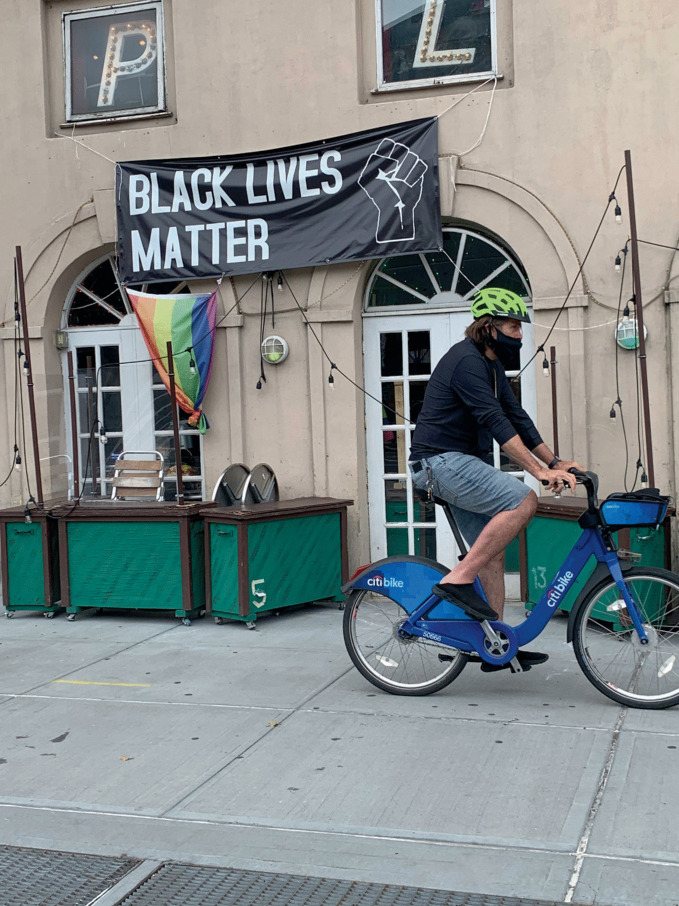
Restaurants and bars in gay neighborhoods closed temporarily and adapted to COVID-19 safety precautions (*Source* Image by Anne Hanavan)

1. *LGBTQ* + *individuals and organizations can participate in “mutual aid networks” to respond to the current* *public health* *crisis, particularly by drawing on community*-*specific knowledge and experience related to the HIV/* *AIDS* *pandemic.*
  The HIV/AIDS pandemic strengthened LGBTQ+ spaces by creating a consolidated urban network which activists used to tackle the ravages of the disease. Similarly, queer communities may once again self-organize by using gayborhoods as a base to disseminate information or distribute face masks and other Personal Protective Equipment (PPE). LGBTQ + communities and organizations leveraged their collective expertise and resources to respond to the HIV/ AIDS pandemic by reducing stigma, encouraging widespread testing, and negotiating better access to treatment (Ghaziani 2008). Despite this well-documented historical example, present-day governments and policy makers have generally overlooked the potential benefits such as grassroots collective activities when considering their responses to COVID-19. For instance, the UK government chose to build centralized laboratories to process tests and rejected an offer to create a complementary network of smaller local providers (BBC 2020a).^1^, ^2^ There remain opportunities for authority figures to work with grassroots organizers, not least because the latter can generate a sense of ownership of health outcomes by (and for) local populations (Marston et al. 2020).
  The potential for action by mutual aid networks within communities, aided by mobile digital technologies, peer-to-peer communication, and a healthy dose of campaigning and activism—all independent of centralized or formalized networks—is already evident in the response to COVID-19 (Butler 2020; Villadiego 2020). The businesses and residents in gayborhoods could build similar movements, attracting external supporters (e.g., visitors and governments) to create a grassroots response to COVID-19 *with their gayboorhood at the center.* In an acceptance speech in June 2019 for the Isabelle Stevenson Tony Award for her outstanding service to the LGBTQ + community, the American actress Judith Light explained that gay men, lesbians, and their allies have a great deal of knowledge about the AIDS pandemic, which they can pass along to other groups and future generations (Bitterman and Hess 2021). She characterized LGBTQ + people as a “community of leadership.” It is our hope that by engaging in their leadership capacity and working together co-operatively—through gayborhood meetings to plan community events, through guerrilla or grassroots advertising campaigns for gay community venues, or through exchanging skills and sharing resources—LGBTQ + communities can capitalize on the strength of their local spaces and sense of communal wellbeing.
2. *New types of LGBTQ*+* institutional anchors may develop within and outside* *gayborhoods* *in response to the pandemic, fostering novel commercial outlets and community spaces.*
  A second unexpected opportunity is that COVID-19 may augment the role of “institutional anchors” (Ghaziani 2014a), distinctive facilities that provide salient markers of urban sexualities (Ghaziani 2021). Consider the recent case of the Little Gay Shop in East Austin, Texas. As recent transplants from New York City, owners Justin Galicz and Kirt Reynolds wanted to open a queer community space that was not centered on alcohol and partying. When you enter this little shipping container on Airport Boulevard, you will find all manner of queer paraphernalia, including t-shirts, pins, patches, books, and magazines. This can be considered an institutional anchor because it provides a place that allows local residents, tourists, and other actors to engage with queer culture, reproducing it through their experiences and enactments.
  Galicz and Reynolds encountered media reports which suggested that LGBTQ + bars, from Austin to New York City, have closed at greater rates as cases of COVID-19 surged. Music festivals, like South by Southwest in Austin, have also been canceled. In light of these challenges posed by the pandemic, the duo realized that a community space like theirs is more important than ever. “They [artists] are people that either freelance or they rely solely on pop-ups and markets and fairs and all of these different types of events to make their living,” Galicz remarked in an interview with *The Austin Chronicle*. “With all of those being canceled or postponed, we felt a responsibility to help them in anyway way we can.” The owners routinely share their proceeds with local queer artistic and political charities.^3^ By extending research on the enduring effects of anchor institutions in the context of assimilation, we argue that new anchors are forming which can provide financial support, cultural offerings, and social opportunities in a pandemic. Unlike other kinds of businesses or organizations, institutional anchors are totems of communal life that represent distinct ways of life. These places, like the Little Gay Shop, can survive even while some bars shutter.
3. *In response to the* *COVID*-*19* *pandemic, LGBTQ* + *groups may migrate from* *gayborhoods* *to other mobilities and settlement patterns.*
  A number of media headlines, such as “ Coronavirus may prompt migration out of American cities” and “Americans flee cities for the suburbs,” suggest that the pandemic has induced a major demographic shift.^4^ But is this shift temporary, or will it have longer lasting effects? In the US, this outbound migration is most pronounced in New York City, where an estimated 5% of the population left the city for a period of time (see Fig. 17.5).^5^ Some headed to vacation homes, while others sought solace with extended family in the suburbs.^6^^,^^7^ The call to shelter in place and a nearly universal mandate to work from home have led some to “predict doom for America’s biggest cities,” while others fear “an urban ice age.”^8^ Even larger migrations have been seen elsewhere in the world, often with more dramatic and disastrous effects. For example, in India the loss of income caused by lockdown forced millions of migrant workers to return to their villages; with transport systems overwhelmed, this reverse migration involved people walking hundreds of miles, with some dying from exhaustion or due to accidents on the road (BBC 2020b). Many countries have attempted to mitigate the socioeconomic effects of these mass movements. In the UK, the government is trying to encourage workers to return to the office (BBC 2020c), effectively attempting to “thaw” the urban economy of restaurants and other services catering to these workers and their clients. However, Florida (2020) predicts that the pandemic will accelerate an attraction to the suburbs for families, while pushing young people and businesses into more peripheral, and thus affordable, urban areas.^9^ See Fig. 17.6.

**Fig. 17.6:**
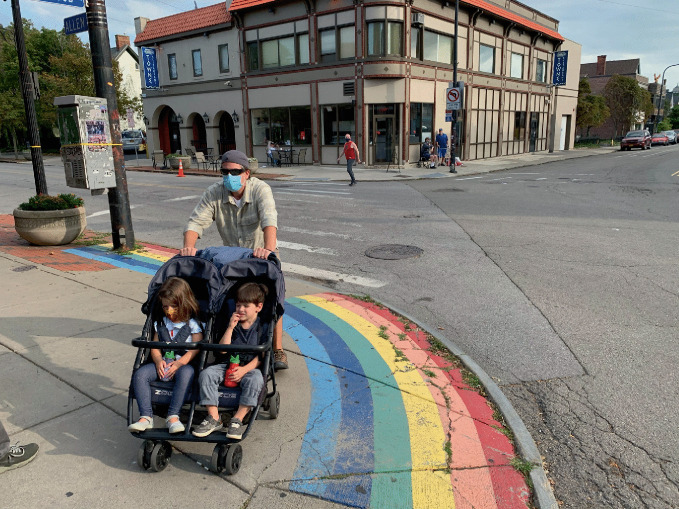
LGBTQ + neighborhoods serve an important function in mid-sized cities, such as the Allentown neighborhood in Buffalo, New York (*Source* Image by Alex Bitterman)
  Although some manifestations of this outward migration are contoured by wealth, like vacation homes, sexuality seems to be a more prominent determinant than class here. These fears are reminiscent of critiques of gayborhood studies for their metronormative emphasis (Halberstam 2005). Not all LGBTQ + people live in cities, and their collective engagement is not exclusively urban. Indeed, scholars have documented diverse spatial expressions and placemaking efforts in peri-urban (Forsyth 1997), suburban (Brekhus 2003; Tongson 2011), and rural environments (Bell and Valentine 1995; Gray 2009), along with ordinary cities (Robinson 2006; G Brown 2008) those in the Global South (Brown et al. 2010). All of these sites are captured by the imagery of cultural archipelagos (Ghaziani 2019a).
  Overlaying pandemic-related population shifts onto scholarly critiques of the gayborhood reveals the power of an expansive analytic gaze that reaches beyond city-center locations. In short: we must shift our focus from urban settlements to wider mobilities (Nash and Gorman-Murray 2014; Hess 2019; Bitterman 2020a). As an example, Bain and Podmore (2020) use Surrey and New Westminster, peripheral metropolitan locations outside the Vancouver urban core, to show how placemaking is a function of resource landscapes (e.g., commercial infrastructure and gathering spaces that residents can use to organize social activities), political opportunity structures (e.g., institutional, policy, planning, and funding frameworks), and inter-organizational relations (e.g., informational, financial, and interpersonal networks). Settlements in places like Surrey and New Westminster may be characterized by less territoriality than downtown Vancouver, and they may lack the critical mass to support the type of institutional density that characterizes the gayborhood, but queer visibility is still undeniable. One respondent asserted with optimism: “that’s what’s unique about New Westminster. We don’t have a village. The whole community is our inclusive village” (Bain and Podmore 2020: 11). In Surrey, NY, which has fewer resources and less political support for LGBTQ + people, “a fragmented and sporadic solidarity” has emerged among its comparatively smaller group of LGBTQ+ residents. In common, the two locales show the importance of using more imaginative measurement protocols to assess placemaking efforts beyond the downtown core of major cities.
4. *Pandemics* *can induce innovations in the configuration and design of interior spaces, which can revive the importance of LGBTQ* + *spaces, especially bars, without compromising* *public health* *protocols and safety procedures.*
  For more than a decade—long before COVID-19 appeared—gays bars have been closing. As many as 37% of gay bars in the US shuttered from 2007 to 2019 (Mattson 2019). Between 2006 and 2016, 58% of LGBTQ + bars, pubs, and nightclubs in London shut down as well (Campkin and Marshall 2017; Ghaziani 2019b). These multi-national findings require us to separate questions about the sustainability of gay bars in general from the specific effects posed by a pandemic. For example, researchers have shown that there were more gay bars operating during the height of the AIDS pandemic than there are today, even before the COVID-19 pandemic. Even though the temporary closures that COVID-19 has induced can (and in some instances already have) become permanent failures, there are still more than 800 gay bars open across 46 US states.^10^ The wisdom here is to focus on an ethos of survival and adaptability—but the question is how to do it.
  During the early months of the pandemic, states and municipalities across the US implemented measures to enforce “social” or “physical distancing,” a public health initiative that has proven to reduce the rate of transmission for respiratory viruses (Lipton and Steinhauer 2020; see Figs. 17.7 and 17.8). By mid-March 2020, more than half of US states issued closure orders for their bars. As the virus spread, some countries, like Spain, Iran, Argentina, Brazil, India, Germany, and Italy placed their citizens on lockdown, a requirement which forced people to “stay home” or “shelter in place.” These measures created a crisis for LGBTQ + communities. Would the novel coronavirus “permanently damage or reshape” urban sexual cultures?^11^ “LGBTQ venues are our own churches. It’s where we form and nurture our community and the individuals within that,” said John Sizzle, a Drag DJ who co-owns The Glory, a queer bar in East London. “Our long-term future is not clear at all.” With voluntary and enforced lockdown sheltering measures in place, many LGBTQ + establishments were operating at significantly reduced capacity. This threatened the ability of owners to pay their rent, utilities, and payroll. “We’ll die!” exclaimed Joaquin Pena, owner of Madrid gay bar Marta Carino.^12^ What does the case of Covid-19 teach us about the mechanisms of LGBTQ + creative resilience?

**Fig. 17.7:**
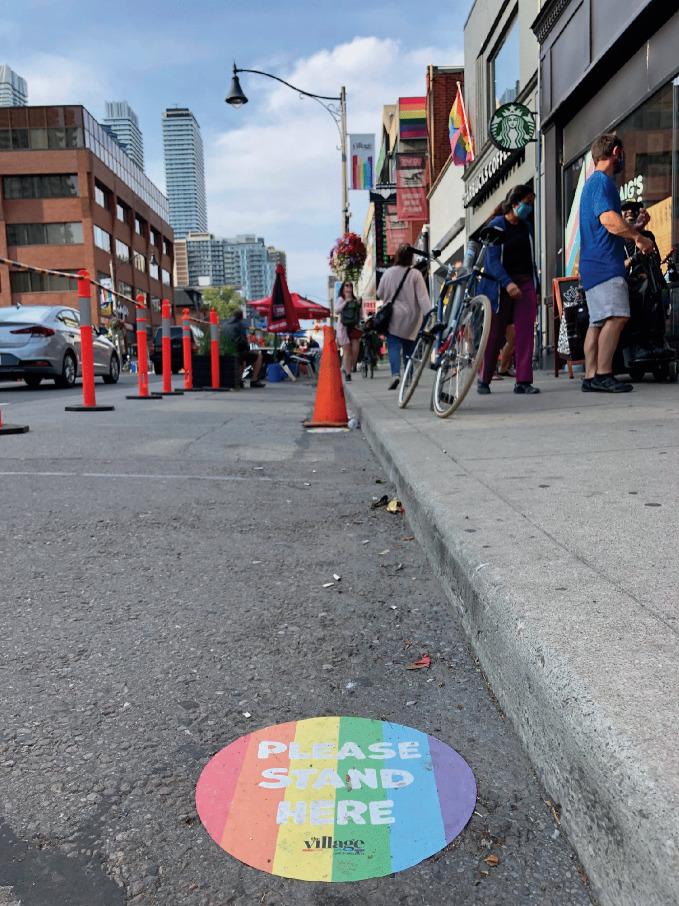
Gay bars, like this one in the Church Street neighborhood of Toronto, added pavement markings requiring patrons to practice spatial distancing in compliance with COVID-19-related regulations (*Source* Image by Rob Modzelewski)

**Fig. 17.8:**
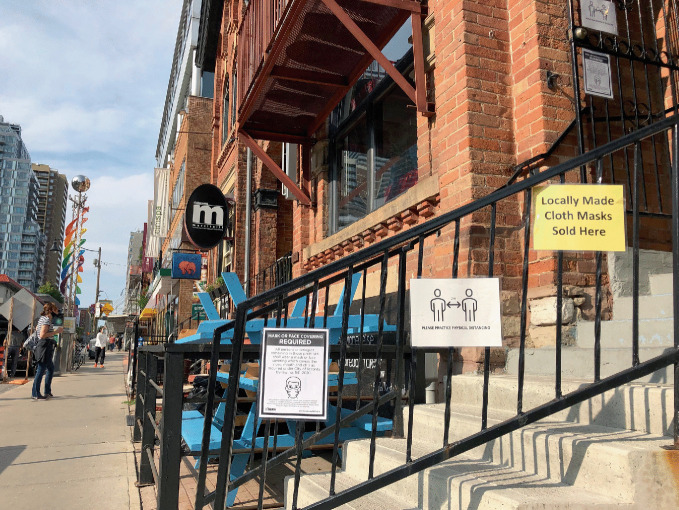
Retail and commercial outlets in the Church Street neighborhood in Toronto remind customers to follow safety measures for the COVID-19 pandemic (*Source* Image by Rob Modzelewski)
  Interior design is a key theme that has enabled bar owners to survive plagues and pandemic. Andersson (2009) shows how gay bars in London’s Soho gayborhood expressed an aesthetic that was designed to counter their stigma as “contaminated” by virtue of their association with AIDS. The result was a very particular look: clean chromed surfaces, “clean” and “hygienic” white walls, minimalist furniture, and youthful “pretty boy” bartenders who projected an image of good health. This aesthetic, which was “profoundly shaped by AIDS” (Andersson 2019: 2994), diffused to gay bars around the world. The style might read today as “ homonormative,” to borrow Andersson’s critique. By tracing its historical origins, however, we can appreciate how then, like today, gay bar owners have used synergies between architecture, interior design, and urban space (Campkin 2007) as a mechanism of creative resilience and adaptive community building in a pandemic.
  The Pumpjack in Vancouver provides a contemporary example. As lockdown restrictions eased in British Columbia, some LGBTQ + establishments considered the possibility of re-opening. According to the protocols established by WorkSafeBC, bars and nightclubs were allowed to do so provided they followed certain safety procedures. Pumpjack was among the first gay pubs—in the gayborhood, no less—to reopen in June 2020. The staff received personal protective equipment, which ensured that guests were served by someone wearing a mask or a face shield. The owner also installed Plexiglas around the bar, along the windows in the front that face the street, and in between the booths. “It’s going to be a new experience,” said Byron Cooke, the general manager. This sentiment of cautious optimism was shared by bar owners across Canada. Dean Odorico, the general manager of gay bar Woody’s in Toronto, added, “The gay community has already lived through a health crisis with AIDS and it brought the community together and it made it a lot stronger…People at the time thought it was the end of days and it definitely wasn’t…The gay community is so resilient.”^13^
5. *The* *COVID*-*19* *pandemic offers new possibilities*—*available to wider audiences*—*for establishing* *virtual communities, not only to replace but also enhance previous ways of connecting.*
  The final generative effect related to the COVID-19 pandemic is a reimagining of the possibilities of the virtual: what it is, how it works, and who feels included within it. In August 2020, *Global News* published the following headline: “The show must go on(line): Vancouver hosts virtual parade amid COVID-19.”^14^ Rather than canceling Pride, as some cities did, the Vancouver Pride Society (VPS) announced that it would shift its celebrations to a series of online events (see Fig. 17.9). “Pride can’t be cancelled,” the non-profit organization declared –“only re-imagined.” Organizers worked tirelessly, and quickly to identify creative alternatives to in-person events. In May, VPS issued a press release: “Vancouver Pride 2020 will go ahead as a virtual reimagining!”^15^ The release outlined a week-long events lineup for the newly dubbed “Virtual Pride 2020.” Highlights included a Virtual Pride Parade, a dedicated day of queer weddings at city hall, and a public (again virtual) disco. VPS also agreed to issue refunds to those who needed them most, and they agreed to pay trans, two-spirit, and queer artists, performers, and musicians as they transitioned their events into a digital space. In a public statement, VPS expressed gratitude, and a generative spirit: “We are so thankful for our Pride family and partners, and for your continued support as we now shift towards creating a different kind of Pride celebration – but one which continues to celebrate diversity and bring us together when we need it most.”^16^ On the day of the event, British Columbia Premier John Horgan tweeted photos of himself at previous parades and wrote, “missing celebrating #VancouverPride in person. But Pride cannot be cancelled.”^17^

**Fig. 17.9:**
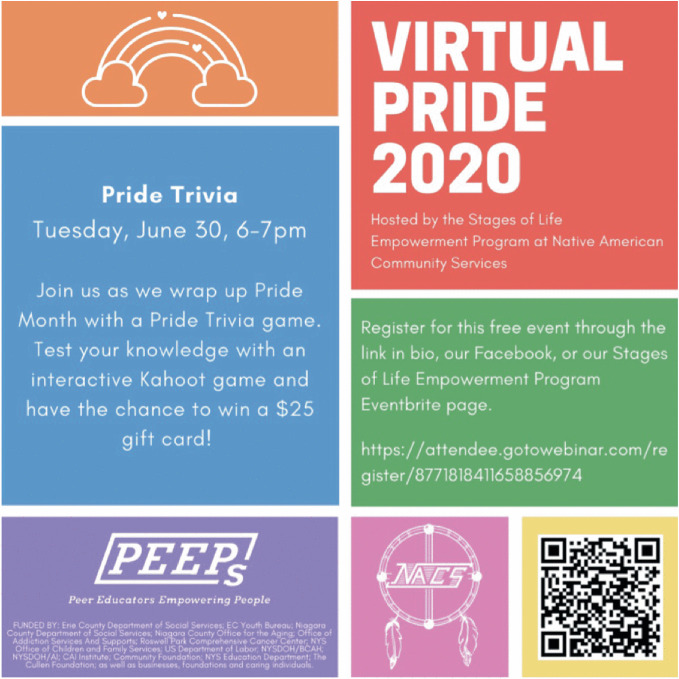
Many LGBTQ+ celebrations in 2020 switched from in-person to virtual events, include the Pride event in Buffalo, New York (*Source* Buffalo Pride Week, used with permission)
  Cities like London, Denver, and Dublin also held virtual Pride events during the summer of 2020. This prompted the *New York Times* to publish an official “2020 Virtual Pride Guide.” The journalist Maya Salam, noted, “LGBTQ Pride events will look and feel very different this year, but many are still on – online.”^18^ Salam acknowledged that “in the era of the coronavirus, traveling and gathering are not options for many. But that should not hinder the spirit and mission of Pride: to remind community members and allies that they are not alone, but part of a greater push for equality, and to elevate the voices and causes central to LGBTQ + people and other marginalized groups.” The global reimagining of Pride—and its inherent symbolism—demonstrates with particular force the resilience of urban queer communities in a worldwide crisis. Whether a virtual pride celebration can capture the same spirit as its physical manifestation remains up for debate. Certainly a crowded, enthusiastic, often sweaty in-person parade becomes impossible, while other elements are compromised or reconfigured, with varying degrees of success. These elements, so key to a physical pride celebration, were no doubt in many cases poorly replicated online in this uncertain year. However, if we recognize alternative potentials of online pride celebrations—whether inclusivity, accessibility, or creativity, of different community members—then there may yet prove to be a generative future for online and hybrid gayborhood community events. See Fig. 17.10.

**Fig. 17.10:**
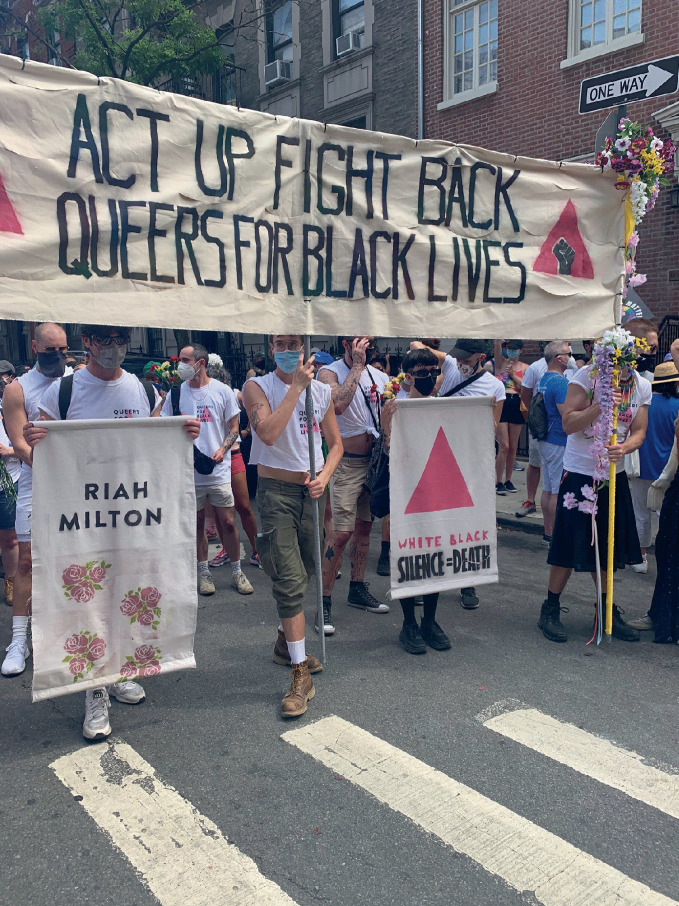
In-person Pride events occurred during summer 2020 in certain cities, including this one in New York City, despite the COVID-19 pandemic (*Source* Image by Anne Hanavan)

## Concluding Remarks: Beyond the Master Metaphor of the Gayborhood?

This edited volume has focused on the past, present, and possible futures of gayborhoods . One critique of the prefix “gay” as a descriptor for these urban areas is that it overemphasizes gay male experience, while potentially erasing others in the “LGBTQ +” acronym. The reasons for strategically retaining “gay” were noted in the first chapter of this book (Hess and Bitterman 2021), but what garners less critical questioning is the latter part of the “gayborhood” portmanteau. In a strict sense, *neighborhood* denotes a spatial concentration of people and organizations whose features contribute to group identity. However, the term also connotes collectivity, togetherness, and many other qualities that are favorably regarded in most cultural contexts. Accordingly, deploying the term “gayborhood” gathers a positive charge, and its potential decline or disappearance is tacitly treated as a negative development. Yet, while the term “neighborhood” certainly directs attention toward certain phenomena, it also delimits other understandings from emerging. This is problematic during a paradigm-shifting event such as a global pandemic. What if something new—something different and as of yet unnamed—is being formed that we cannot adequately capture, or properly consider, by our conventional cognitive schemas about LGBTQ + urban spaces and places? Is it possible that “the gayborhood” is no longer the most appropriate archetype for discussions about LGBTQ+ spatiality in a post-COVID-19 era?

Spatial metaphors abound in studies of sexuality and gender. Take “the closet,” a concept interrogated by Sedgwick (1990) and also immediately recognizable to LGBTQ + people and heterosexuals alike. The closet creates an internal space with connotations of claustrophobia and containment, but also safety and comfort, depending on the circumstances (Pantazopolous and Bettany 2010). For years the emphasis was on the negative, with the assumption that everyone wants to “come out” if they can (Cass 1979). Yet, familiarity with a restrained space like the closet might help in the era of COVID-19. After all, what is home quarantine if not a larger closet?

**Fig. 17.11:**
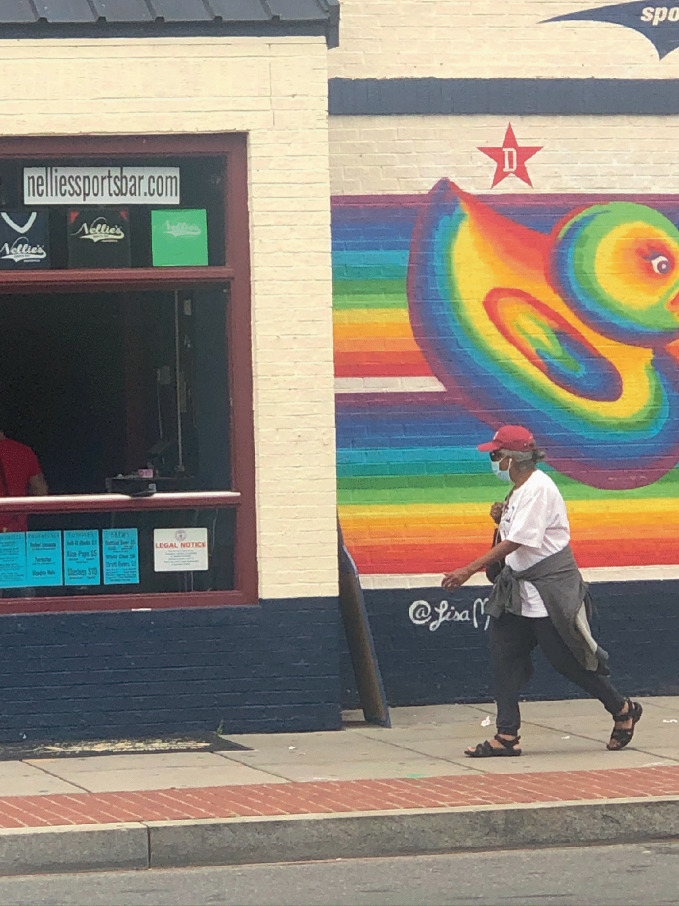
In LGBTQ + neighborhoods, residents and visitors complied with the safety precautions required by the COVID-19 pandemic (*Source* Image by Elizabeth R. June)

**Fig. 17.12:**
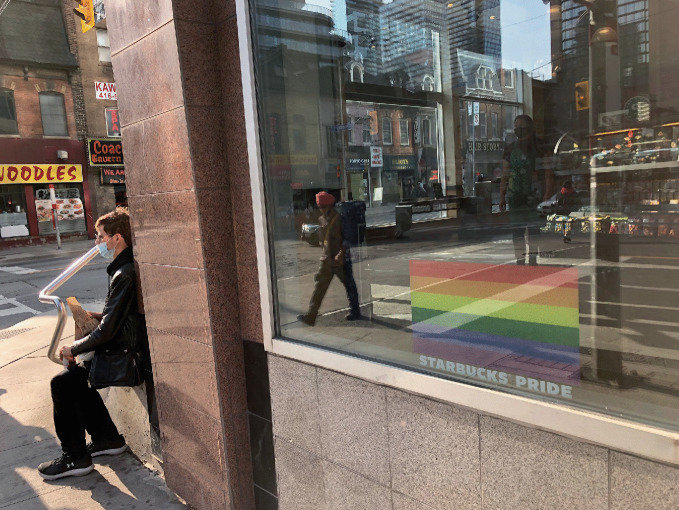
Retail and commercial outlets in gay neighborhoods adapted to COVID-19-related regulations (*Source* Image by Rob Modzelewski)

The relationship between the closet and lockdowns may actually be closer than initially conceived. National and local lockdowns have been used to slow the transmission of COVID-19. Although the specifics vary, lockdowns usually involve restricting movements and activities (see Fig. 17.11). In extreme cases, people are locked down at home or in another domesticated space (e.g., a hotel room), with commercial premises closed and access to public places constricted. See Fig. 17.12. Evidently, lockdowns have a huge impact on the viability and vitality of most non-residential sites including, as noted above, gayborhoods. Most commentators may conclude that lockdowns are a temporary phenomenon, soon to be resigned to the annals of history as a peculiar feature of 2020. However, as lockdowns continued into 2021, and if they are reinstated in response to future pandemics, they likely will emerge as a new socio-spatial archetype in the collective consciousness. By this we mean that lockdowns might form a way of thinking about social and spatial relationships that transcends particular historical and geographical contexts, just as the term “ghetto” has left its origins in Venice to be applied to a variety of contexts (Ghaziani 2015; Coffin et al. 2016). Might young LGBTQ + people grow up thinking of their dynamic coming out experiences as varying degrees of lockdown, rather than time spent in a closet?

**Fig. 17.13:**
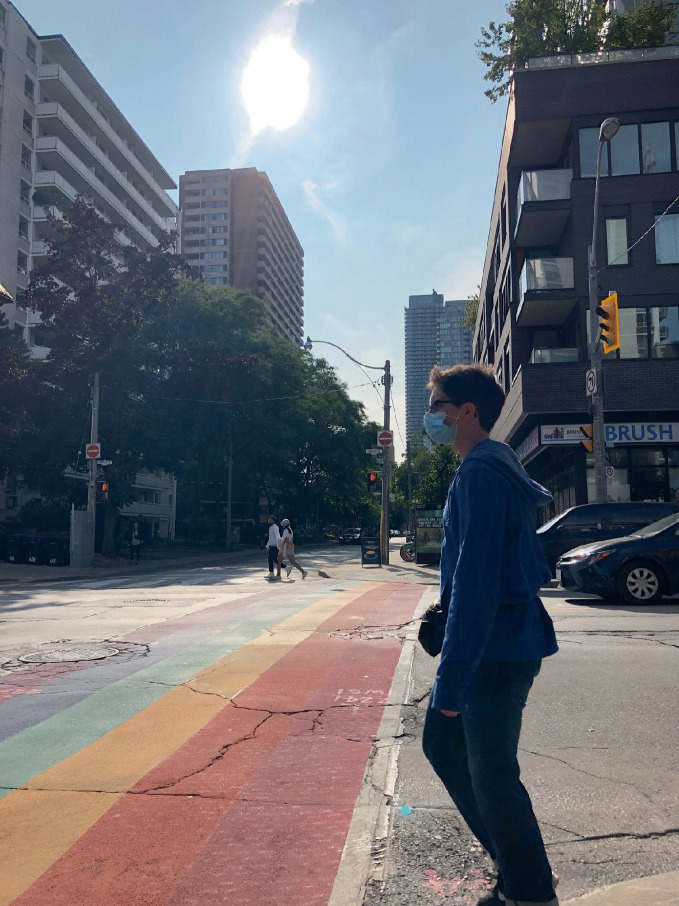
Even with safety precautions in place due to the COVID-19 pandemic, LGBTQ + neighborhoods can provide a welcoming space for everyone (*Source* Image by Rob Modzelewski)

Then again, those who have yet to disclose their non-heteronormative identity may be under increased scrutiny when they are sharing a small space with significant others for extended periods of time. Lockdown conditions may make the closet more claustrophobic than ever (see Fig. 17.13). Since the outset of the pandemic, acute mental distress and suicidal ideation has been alarmingly high for adolescents and young people, but for LGBTQ + youth who are forced to move from the relative freedom that schools, colleges and houseshares offered back to their childhood homes, mental health is drastically affected. “ Re-closeting” their sexuality—to transition from visibility to invisibility and silence once again—to their families, who may be conservative, unaccepting, vehemently opposed to the individual’s sexuality or indeed unaware of it, is incredibly distressing (Batty 2020a, b; Dasgupta 2020; LGBT Foundation 2020; Kneale and Bacares 2020). The conceptual upshot of this is that lockdown may need to be reconceptualized. It is not simply a temporary phenomenon but perhaps an emergent frame that shapes the experiences of LGBTQ + people. As such it warrants further consideration as a complement to, if not evolution of, the closet concept.

Living alone may alleviate the claustrophobia of (re-) closeted lockdown, but brings with it the potential for isolation, loneliness, and withdrawal. One solution is to escape into online spaces, which leads to a second spatial metaphor that is commonplace in contemporary discussions. In material terms, the Internet is a complex network of cables, satellites, and mobile devices. Figuratively, however, it is a series of *sites* that one can visit, explore, and inhabit for a time. Consider terms like *internet forum*, *online marketplace*, and *virtual waiting room*. Space is the foundational, albeit often implicit, figure of thinking for digitally mediated subjectivity (Miles 2021). Might the over-spatialization of internet interactivity preclude alternative understandings of how electronic technologies enfold into physical realities (Coffin 2021)? For example, smartphones suggest that the internet is no longer a separate topology of virtual spaces but rather another layer of spatiality overlain unevenly onto physical topographies (Šimůnková 2019). Devices are tracked in real-time and also real space, with dating applications often organizing users by distance, and customer reviews allowing photographs and descriptions to be viewed before a physical place is encountered in the flesh.^19^ Thus, technology not only generates new spaces in the imagination (e.g., purely virtual fora), but it also distorts physical spatial experiences (i.e. decreasing the possibility of “direct” or unmediated experience of place). What term can replace the dichotomy between offline and online space? We propose the term *topology*, which invokes a sense of space defined by its dynamic relations rather than fixed physical or abstract features (Cresswell 2013).

 Topologies link back to lockdowns, insofar as technology also allows people to communicate online and then meet in physical locations. During times of lockdown, people may meet at each other’s homes, but in less restrictive phases people may meet in public places or commercial premises. Technologies certainly facilitate clandestine practices such as cruising in public parks, which are affectively charged with a sense of danger during a pandemic, but also allow any commercial site to become a temporary LGBTQ + meeting place. If socio-spatial relationships become more fluid, thanks to the interaction of technological affordances and lockdown restrictions, then perhaps topology is the most appropriate trope through which to think about the future of LGBTQ + cultures. Certainly the flexible and fleeting metaphor of topology should be complemented by other concepts. For instance, while many sites may be fleetingly “queered” the term “archipelago” remains apt to describe the more stable landscape of regularly used and explicitly identified LGBTQ+ sites (Ghaziani 2019a). Similarly, terms like circuit (Ghaziani and Cook 2005), scene (Ridge et al. 1997; Taylor 2010) and pop-up (Stillwagon and Ghaziani 2019) may be useful to describe particular topological formations for LGBTQ + people. Indeed, we celebrate metaphorical multiplicity, as it may draw attention to a greater range of differences between non-heterosexual people. However, we propose that topology is particularly productive as an agnostic or umbrella term, insofar as it can also be transferred beyond the specific context of post-pandemic queer spatiality. In doing so, it can articulate the unique contingencies of *queer* topologies but also their connections to the shifting spatialities of *BAME* topologies, *feminist* topologies, *religious* topologies, and the like. Thus, it is apposite to conclude this chapter, and the edited volume, with a pregnant proposition: the life and afterlives of gayborhoods is the conception of plural queer topologies.

## Footnotes

1 Sir Paul Nurse, the chief executive of the Francis Crick research institute, described the proposed approach using the analogy of the Dunkirk evacuation in World War.

2 The government has put some big boats, destroyers in place. That’s a bit more cumbersome to get working and we wish them all the luck to do that, but we little boats can contribute as well.

3 https://www.statesman.com/entertainmentlife/20200720/little-gay-shop-opens-in-east-austin-with-art-books-and-big-dreams; https://www.austinchronicle.com/columns/2020-07-31/qmmunity-the-best-little-gay-shop-in-austin/.

4 https://fortune.com/2020/07/17/people-leaving-cities-coronavirus-data-population-millennials-marriage-families-housing-real-estate-suburbs/.

5 https://www.nytimes.com/interactive/2020/05/16/nyregion/nyc-coronavirus-moving-leaving.html.

6 https://www.theatlantic.com/health/archive/2020/06/pandemic-cities-moving-remote-work/613069/.

7 https://www.zillow.com/research/2020-urb-suburb-market-report-27712/.

8 https://www.theatlantic.com/health/archive/2020/06/pandemic-cities-moving-remote-work/613069/.

9 https://www.bloomberg.com/news/features/2020-07-02/how-coronavirus-will-reshape-u-s-cities.

10 https://slate.com/human-interest/2020/05/gay-bars-struggling-reopen-coronavirus.html.

11 https://www.theatlantic.com/culture/archive/2020/06/how-quarantine-reshaping-queer-nightlife/612865/.

12 https://nationalpost.com/pmn/entertainment-pmn/well-die-gay-bars-risk-coronavirus-collapse.

13 https://www.vice.com/en_ca/article/qj4bgx/queer-nightlife-will-find-a-way-through-covid-19.

14 https://globalnews.ca/news/7246594/coronavirus-vancouver-virtual-pride-parade/.

15 https://www.insidevancouver.ca/2020/05/19/vancouver-pride-2020-will-be-virtual/.

16 https://dailyhive.com/vancouver/vancouver-pride-parade-cancelled-virtual.

17 https://www.citynews1130.com/2020/08/02/vancouver-pride-virtual-parade/.

18 https://www.nytimes.com/article/gay-pride-2020-events-online.html.

19 As one illustrative speculation: will gay bars and other LGBTQ + commercial environments receive a crowd-sourced “cleanliness” or “hygiene” rating and, if so, how might the added business costs and consumer concerns shape the ongoing viability of those already struggling to survive?
